# Nonadherence Predictors to Tuberculosis Medications among TB Patients in Gambella Region of Ethiopia

**DOI:** 10.1155/2022/9449070

**Published:** 2022-08-24

**Authors:** Taye Kebede, Wiw Gach Jing, Abiot Girma, Kifle Woldemichael

**Affiliations:** ^1^Department of Biomedical Sciences and Immunology, Natural Sciences College, Madda Walabu University, P.O. Box 247, Bale-Robe, Ethiopia; ^2^Aklilu Lemma Institute of Pathobiology, Addis Ababa University, P.O. Box 1176, Addis Ababa, Ethiopia; ^3^Division of Epidemiology, Gambella Regional Health Bureau, Gambella Region, P.O. Box 408, Gambella, Ethiopia; ^4^Department of Public Health, College of Health Sciences and Medicine, Jimma University, P.O. Box 378, Jimma, Ethiopia; ^5^Department of Epidemiology, College of Health Sciences and Medicine, Jimma University, P.O. Box 378, Jimma, Ethiopia

## Abstract

**Background:**

Global tuberculosis (TB) disease deaths rise comparable to the one seen in 2015 (by 200,000) or even in 2012 (by 400,000) as a result of the potential impact of the COVID-19 pandemic. Ethiopia's Gambella region is leading for years in terms of TB cases and its comorbidities. The TB control program effectiveness depends on in large on the patients completing the appropriate treatment regimen. Hence, the objective of this study was to determine the determinants of nonadherence to anti-TB drug treatment among patients in Gambella regional state of Ethiopia.

**Methods:**

A case-control study was conducted on cohorts of TB patients sampled from four public health facilities in Gambella Region from January 2019 to 2020, followed by 18 months of follow-up. The total sample size was 296 patients (74 cases and 222 controls) with a response rate of 97.3% (288 : 72 nonadhered cases and 216 controls). Cases (nonadhered) were TB patients who missed 10% of the doses while controls were patients, who completed 90% or more doses.

**Results:**

TB patients, who perceived stigma [AOR = 2.7 at 95% CI (1.1–6.6) with *P* value <0.05], failed to receive any counseling during the treatment course [AOR = 65.24 at 95% CI (11.69–363.95) with *P* value <0.01], patients who used to smoking during treatment [AOR = 15.4 at 95% CI (7.7–30) with *P* value <0.01], taking TB medications regularly has no benefits [AOR = 6.8 at 95% CI (1.8–24.9) with *P* value <0.05], and patients believing TB disease as not severe [AOR = 8.38 at 95% CI (2.0–34.6) with *P* value <0.05] were significantly or highly significantly associated with nonadherence to anti-TB drugs medications.

**Conclusion:**

The determinants of nonadherence to anti-TB treatment among TB patients in the Gambella region during the study period were the absence of counselling services, and patients' behavior (smoking habits, undermining the severity of TB disease, lack of trust in the outcomes of regular medications, and perceived stigmatization). Accordingly, capacitating healthcare providers and workers at all TB clinics for effective counseling , preventing perceived stigma by protecting the patient's secrecy, and routine health education has paramount importance for effective TB control in Gambella.

## 1. Introduction

Tuberculosis (TB) is an infectious disease caused by *Mycobacterium tuberculosis*. Pulmonary TB is the most common form that mainly affects the lungs. Sporadically, the disease can be caused by *Mycobacterium bovis* and *Mycobacterium africanum*. Pulmonary TB (PTB) is transmittable. Any form of TB involving nonlung organs such as the pleura, lymph nodes, spine, joints, genito-urinary tract, nervous system, glands, and abdomen is considered extra pulmonary tuberculosis (EPTB) [1, 2].

Tuberculosis is a global public health concern. According to the WHO report, the years 2012 and 2015 were the two worst scenarios in the modern history of TB disease globally. In 2015, the disease accounted for an incidence of 10.4 million TB cases. Out of this, 10.4 million, 5.9, 3.5, and 1.0 million new TB cases were men, women, and children respectively [3]. In addition to this; an estimate of 480,000 new cases of multidrug-resistant TB (MDR-TB) were reported worldwide in the same year with an additional 100, 000 people with rifampicin-resistant TB (RR-TB), who were also newly eligible for MDR-TB treatment [4]. Furthermore, Human Immunodeficiency Virus (HIV/AIDS) exacerbated the condition by increasing susceptibility to TB infection and progression to disease; facilitating the reactivation rate and increased reinfection rate [5]. Although many countries' efforts to achieve the Millennium Development Goals (MDGs) TB plans somehow lowered the TB burden, many scholars still strongly argue that the TB spillover effects can be obstacles to achieving Sustainable Development Goals (SDGs) 5 (gender equality), and SDG 10 (reducing inequalities) set prospectively [6].

Tuberculosis is a major economic setback for many developing regions of the world [7]. In Ethiopia, tuberculosis is a leading cause of death among communicable diseases [8]. The outcomes of TB treatment are categorized as cured, treatment completed, treatment failure, died, defaulters, and transferred out where treatment outcome is unknown [9]. The treatment success rate is below the global target (85%), which jeopardized the amenability of MDG [10], where the failure of treatment continuation is among the main possible reasons. In Ethiopia, the TB cure rate is estimated to be 67% [11, 12]. The regional treatment success rate and follow-up of TB patients are haphazardly monitored at the grass-root level, where strict DOTS implementation is still capable of reducing the burden of death and economic losses [11].

In the year 2019 REPORT, among the 22 highest TB-burdened countries, Ethiopia ranks third in Africa and eighth in the world [12]. Tuberculosis kills an estimated 32,000 Ethiopians every year (more than 80 people per day), and has a long-term corrosive impact on the health of the Ethiopian citizens [13]. Tuberculosis in Ethiopia mostly affects the productive young age group. For instance, 58% of TB prevalence was reported in those under 35 years of age. A significant proportion of 32,000 deaths per year was indicated in young adults [14]. The economic consequences of TB include those who lost income and productivity during diagnosis and treatment; direct household expenditures for TB care; and unmeasured disabilities due to permanent lung damage in up to 50% of survivors. Since the majority of TB cases occur among young adults and children, it heightens an economic cost [15].

It is before a decade that the WHO report warned the status of TB in the Gambella Region of Ethiopia as the highest among all regional states of the country, which needs special attention with the notification rate (new and relapse) of 261 to 421 per 100,000 people [16]. To this effect, the newly adopted Sustainable Development Goals (SDGs) demand to achieve of universal health coverage in which effective treatment for all TB-infected people has been stipulated under its target. In this regard, TB treatment coverage is one of the top priority indicators to monitor the progress toward the End TB Strategy by 2030 and 2035 [17].

Treatment nonadherence is the inability to obey the protocols of treatment. Specifically, treatment adherence in terms of TB control is defined as the extent to which the patient's history of therapeutic drug taking coincides with the prescribed treatment [18]. Hence, adherence to TB medication is an area of concern. The national strategy is aligned with the global TB end strategy to end the TB epidemic by 2035 [17]. On top of the active surveillance and prevention of TB, medication adherence is one of the crucial factors in the realization of a TB control program [19]. On the other hand, nonadherence to treatment prolongs the illness and enhances transmission and death [20]. Inappropriate treatment and irregular use of anti-TB drugs trigger the spread of MDR-TB strains, an emerging public health threat worldwide [21]. Likewise, the improper treatment regimen for MDR-TB is a fertile ground for the development of extensively drug-resistant TB (XDR-TB) [20, 21].

Effective treatment of all forms of TB cases with rigorous adherence to medication is one of the key TB intervention strategies. However, barriers to adherence are complex and need multifactorial remedies [22]. TB patient commitment and abilities are linked to various determinants; such as patients' own factors, social and economic factors, healthcare workers (HCWs) factors, the health system, characteristics of the disease, and disease therapies [23]. Consequently, addressing the gap in TB patients' nonadherence to treatment is crucial for the welfare of the community as TB requires a long duration of treatment [24].

In Gambella regional state, a few TB treatment outcomes and associated factors studies have been made so far [16]. Hence, the study in this regard in Gambella regional state (the region which leads in terms of TB burden and its comorbidities in Ethiopia) by reaching each TB patient household in person in the districts of the region has paramount importance for the country. Hence, the current study aimed to assess the determinants of nonadherence to anti-TB drug treatment among TB patients in the districts of Gambella regional state, Ethiopia; where patient-related, HCWs, and healthcare provider-related factors were evaluated in detail through a case-control study.

## 2. Materials and Methods

### 2.1. Study Area and Period

Gambella is one of the regional states in Ethiopia, located in the western part of the country. The region coordinates are 7.9220°N and 34.1532°E. According to the 2017 population projection, the region has an estimated total population of 435,135 (226,839 males and 208,296 females) [25]. The region is one of the areas that suffers from a repeated influx of refugee population from unstable neighbors. It shares a long border with South Sudan and two other Ethiopian regions; Oromia to the north and east and the Southern Nations, Nationalities, and Peoples' Regional State (SNNPRS) to the south. The region has an area coverage of 29,783 km^2^. The topography divides the Gambella region into two broad subregions, which are between 900 and 2,200 m a.s.l. (meters above sea level), and flood plains below 500 m a.s.l. The Gambella region lies within the hot to warm humid lowland agro-ecological zone. Its climate is classified as tropical savannah with an average temperature of 27.6°C (varies from 21.1°C to 40.9°C). In the lower altitudes, the annual rainfall varies from 900 to 1,500 mm, while at higher altitudes it ranges from 1,900 to 2,100 mm. The rainy season starts at the end of April and lasts in October, with the maximum rainfall in July [26].

Gambella is divided into three zones, namely, Nuer, Agnuwuak, and Majang. The formation of zones is based on the majority tribal population number that inhabits the specific localities. Collectively, the region has 13 districts, one special district (Itang), and 158 Kebeles. The regional capital is Gambella city. The major rivers in the region are the Baro, Akobo, Alwero, and Gillo [27]. Gambella region has a regional hospital, three district hospitals, and twenty-three health centers that provide TB diagnosis and treatment services in the region. On top of these, another five health centers provide TB diagnostic and treatment services to the refugee population in the area. Although more than 50 private health facilities are operating in the region, none of them address TB-related services. Until recently, to our knowledge, there is a high shortage of a fully-fledged public health institution serving as an MDR-TB treatment initiation hub in the region [28].

### 2.2. Study Design

A case-control study was conducted among TB patients of all forms (pulmonary smear-positive to smear-positive and negative) as well as extrapulmonary TB. Cases and controls were obtained from the lists of TB patients at health facilities during the study period based on the preset inclusion and exclusion criteria. Cases (nonadhered) and controls (adhered) were defined based on the missed days (doses) during the entire treatment period. The definitions of cases and controls were adopted from a previous study conducted in the Tigray region of Ethiopia, where nonadhered (cases) were stated as those TB patients who missed at least 10% of doses while adhered (controls) as those TB patients, who completed at least 90% of the recommended doses throughout the treatment period [29].

### 2.3. Source and Study Population

The source population of the study was all patients, who had followed TB treatment from January 2019 to 2020 in four public health facilities in the Gambella region that deliver TB treatment services in the region. The recommended standard treatment schedule for all forms of TB cases (PTB and EPTB) is six months except for MDR-TB patients, which require 18 or more months. However, some forms of EPTB, which involve the central nervous system (CNS), TB meningitis, musculoskeletal TB, and TB pericarditis, require 12 months of treatment duration [30, 31]. On the other hand, the study population was all TB patients, who fulfilled the inclusion criteria, sampled and followed, coming from all corners of the Gambella region, who were attached to Gambella Hospital, Punyido Health center, Kuergeng Health Center, and Nyinenyang Health Center for TB treatment during the study period.

### 2.4. Inclusion and Exclusion Criteria

All TB patients aged 18 years or above were included in the study after a pilot study had been made. The age limit was based on the legal age of informed consent for participation in research activities. It is clearly stated under National Research Ethics Review Guideline in Ethiopia as a legal age indicator for nonminors, who have a full right to give informed consent and willingly participate in research activities [32]. The exclusion criteria were TB patients previously diagnosed with MDR-TB patients, TB patients previously categorized under treatment failure, TB patients, whose contacts have already been lost and difficult to contact, TB patients, who had changed their addresses or transferred to another unit, and TB patients, who were mentally and physically unstable to give a proper response.

### 2.5. Sample Size Determination

The sample size of the study was determined using the formula for calculating an independent case-control study (unmatched case-control study) [33]. Furthermore, to minimize the manual calculation errors, the sample size was calculated by an automated tool using Epi Info 7-Stat Calc designed for calculating sample size and power [34].

(1) $$\begin{array}{c} n=\frac{{\left(Z\alpha +Z\beta \right)}^{2}\times 2P\left(1-P\right)}{{\left(P1-P2\right)}^{2}}\times \frac{\left(C+1\right)}{2C}, \end{array}$$

(2) $$\begin{array}{c} P=\frac{P1+P2\;}{2}, \end{array}$$

(3) $$\begin{array}{c} P1=\frac{P2*r}{1+P2*\left(r-1\right)}, \end{array}$$

where *n* is the number of cases (*n*); (*r*) is the odds ratio (odds ratio of exposures between cases and controls), and (*c*) is the number of control subjects per case subject which were set as 3. This is because the most efficient ratio of case to control is 1, but the cost or design considerations dictate the use of a different ratio. Particularly, for greater generalizability, multiple control groups are used in the study. However, beyond 4 controls per case does not improve precision [35]. While (*cn*) is the number of controls; (*n* + *cn*) is the total sample size; (*Zα*) is the desired level of statistical significance at 95% confidence; (*Zβ*) is the desired statistical power at 95%; (*P*1) is the proportion of exposure among cases; (*P*2) is the proportion of exposure among controls; and (*P*) is the average exposure between the case and control subjects.

Although a single district, a previous study conducted in the Itang special district of Gambella region reported 42% of the participants from the district community had a poor knowledge score about TB disease [36]. Hence, in our study, the exposure level among the controls was estimated as a population prevalence of exposure (*P*2), which was estimated to be 42% (0.42). On the other hand, the odds ratio (*r*) of exposure between cases and controls was estimated to be 2.72 from another previous study conducted in the Tigray Regional State of Ethiopia, which reported that TB patients who had felt ashamed or stigmatized were 2.72 times more likely to be nonadherent to anti-TB drugs treatment as compared to those who did not [29].

As a result, *P*1=(*P*2*∗* *r*)/(1+*P*2*∗*(*r* − 1)).

By substitution, *P*1=(0.42*∗*2.72)/(1+(0.42)*∗*(2.72 − 1).

(4) $$\begin{array}{c} P1=\frac{1.1424}{1.7224}. \end{array}$$

*P*1=0.66 (66%), which is the exposure prevalence among cases.

(5) $$\begin{array}{c} q1=0.34. \end{array}$$

The value of *P*=(*P*1+*P*2)/2, an average of *P*1 and *P*2.

*P*=(0.66+0.42)/2 = 0.54, the average exposure rate between the cases and controls.

*Q* = 0.46; consequently, the sample size in the case group (nonadherent) was determined as

(6) $$\begin{array}{c} n=\frac{{\left(Z\alpha +Z\beta \right)}^{2}\times 2\times 0.54\left(1-0.54\right)}{{\left(0.66-0.42\right)}^{2}}\times \frac{\left(3+1\right)}{2\times 3}=74. \end{array}$$

Thus, the number of controls is (*cn*) which is equal to 74 × 3 = 222, which makes the total sample size (*n* + *cn*) equal to 74 + 222 = 296 individuals, with a zero-nonresponse expectation (emanated from the enthusiastic experience gained during the pilot study at the health centers of refugee camps in the same region). Although not bad, during the actual study data collection period, we encountered only a 2.7% nonresponse rate (2 nonadhered cases and 6 individuals from the control group).

### 2.6. Sampling Procedures

Four public health facilities were purposively selected based on the TB patients load of the Gambella region. Proportional to population size ratio and allocation were made to allocate the number of cases and controls to each health facility (Table 1). Both cases and controls were selected using systematic random sampling techniques from the list of TB patients after the first case/control was selected by a simple random sampling method. Since the study subjects were at home after completing the continuation phase, they were traced using their contact addresses/telephone numbers found in their treatment archives in anti-TB treatment units.

(*n*) Is the number of cases required, (*c*) is the number of controls per case, (*c*^*∗*^*n*) is the number of controls, [(*n*+(*c*^*∗*^*n*)] is the total number of sample size, and (*P*) is the TB patient load in each health facility.

The number of cases was selected using the proportional allocation of a sample size to the population of patients in each facility and calculated by *ni*=*pi∗n*/*Tp*.

Here, (*ni*) is the number of cases required from each facility, (*pi*) is the TB patients loaded in each health facility, (*Tp*) is the total number of TB patients loaded in four health facilities (third-quarter report from Gambella Regional Health Bureau in the year 2018), and (*n*) is the total number of cases calculated from the proportionally distributed load among each health facility (*n* = 72).

### 2.7. Study Variables

The outcome variable of this study is nonadherence, while the rests are independent variables related to socio-demographic, socio-economic, patient-behavior related, HCWs-related, healthcare provider-related, and disease characteristics and complexity of treatment regimen-related factors.

The socio-demographic factors are age, gender, ethnicity, religion, and marital status; whereas the socio-economic factors are income, occupational status, educational status, and family support for the patient. The patient-behavior related factors are knowledge about TB disease and its treatment duration, perception about the cost related to TB disease, perceived susceptibility to MDR-TB, perceived severity of TB, beliefs about modern TB medicine, beliefs about the benefit of regular medications, perceived stigma, forgetfulness, residence (urban and rural), alcohol use, substance abuse, and smoking status. The HCWs-related factors are instructions on how to take medicine, information on the importance of treatment, information on side effects and consequences of stopping medicine, protecting the privacy of the patients, and addressing the emotional and spiritual suffering of the patient during counseling . The healthcare provider or system-related factors are availability and accessibility of the drug, treatment strategy (DOTS and home-based), waiting time for receiving the drug, and distance from the healthcare provider. Finally, the disease characteristics and complexity of the treatment regimen are drug side effects, drug burden, and duration of treatment.

### 2.8. Data Collection Procedures and Instruments

Graduating class students of Gambella University, from related disciplines, were recruited on a competitive basis and trained for data collection before the actual data collection period. Additionally, the data collectors were tested for their practical skills during the pilot study period in health centers serving refugee communities in the Gambella region. Again, knowledge of the local language was given due attention as prohibiting language barriers helps to harvest the real intention and response of the TB patients. As a result, the data collectors are fluent in at least one of the two dominant languages of the Gambella region.

Two-day theoretical training in Gambella city and five days of practical sessions in no sampled health centers were given to the data collectors. The training covered the following topics; general knowledge of TB disease and treatment as per the national guidelines, TB control program, objectives and importance of the study, and detailed orientation of the questionnaires. Communication skills were incorporated to obtain a piece of reliable information from the study participants. The pretesting of the questionnaires was conducted on nineteen TB patients who were linked to the DOTS package. The TB patients for the pilot study were contacted at their own homes for the detailed interview. The results were analyzed manually to check the consistency of the questionnaires. Then, important modifications were made to the tools introduced based on the pretest results.

A semistructured questionnaire was employed as a tool for collecting data through face-to-face interviews with TB patients. The questionnaire was prepared in English and translated into five local languages commonly spoken for communication in the region (Nuer, Agnuwuak, Majang, Afaan, Oromo, and Amharic), and back-translated to English. The translation from English to local languages and vice versa was made by employing an experienced University instructor from the college of language studies of Madda Walabu University (for Afaan, Oromo, and Amharic), and Gambella Region Tourism and Cultural Office (for Nuer, Agnuwuak, and Majang).

### 2.9. Data Quality Control

Training of data collectors and pretesting of the questionnaire had significantly contributed to the data quality assurance. Moreover, the completeness and consistency of the data were checked in the field by the data collectors, which were then strictly monitored and verified again by the researchers (supervisors) at the end of every other day of sessions. Double entry of the data into Epi Data version 3.1 was performed to minimize the errors during the data entry time. We believe that the degree of precision of the data was also enhanced by engaging and recruiting young energetic, enthusiastic graduating class university students. Finally, before data analyses, the missing and duplication of the data were thoroughly executed.

### 2.10. Data Management and Analysis

After completion of the data entry, data were exported to SPSS version 22. For categorical variables; bivariate analyses or cross-tabulation of each independent variable with the outcome variable was performed using the *χ*^2^ test. Fisher's exact test for two-way tables was applied whenever a cell value was below 5. The criterion for statistical significance was set at a *P* value less than 0.05 with a two-tailed test. All variables that were not significantly associated with the outcome variable by the *χ*^2^ test were not recruited for bivariate analysis.

All independent variables that have a significant association with nonadherence status were analyzed using bivariate analysis. All variables with a *P* value less than 0.25 were qualified as a candidate for the multivariable logistic regression model. The use of the *P* value 0.25 as a screening criterion was adopted from the work of Bendel and Afifi [37] on linear regression and the work of Mickey and Greenland [38] on logistic regression. These authors argued that the use of a significance level at a *P* value less than 0.05 often fails to identify important variables. After the completion of the bivariate analysis, multicollinearity among the variables, which qualified for multivariable logistic regression was diagnosed and checked by considering the Pearson correlation coefficient (*r*), where the cutoff point was that *r* = 0.8, and the abovementioned data indicate multicollinearity, otherwise, no concern [39]. In such biomedical statistics, whenever two or more variables have a multicollinearity effect, one of them was removed to reduce redundancy.

Then a multivariate logistic regression model was built using backward LR by removing all insignificant until a simple model with main predictors was obtained. Adequacy of the model to fit the outcome variable with the predictors was checked using the Hosmer and Lemeshow et al. test for the goodness of fit. With a *P* value less than 0.05, the model would not fit the data very well. Otherwise, it would be adequate to fit the data [40]. The model has indicated the goodness of fit (Hosmer and Lemeshow test) at *P* value = 0.725). Finally, the interaction terms were added to the model containing all main effects, and their significance was assessed using the likelihood ratio test. Then the two models were compared to check the effect of interaction terms.

### 2.11. Operational Definitions

The following operational definitions are given for the current study based on the standard definition given by WHO and the Centers for Disease Control and Prevention of the United States of America (CDC) [41, 42]:

  Nonadherent: TB patients who missed 10% of the doses during the duration of the treatment. For the six months treatment period, 10% of the doses are equivalent to 18 days. Meaning that 10% of 180 days of the entire treatment duration. For 12 months duration of treatment, 10% of the missed doses are equivalent to 36 days. This information can be found in the patients' records at TB treatment units.
  Adherents: TB patients who completed 90% or more of the recommended doses during the entire duration of the treatment.
  Smear-positive pulmonary TB (PTB+): patients with at least two initial sputum smear examinations positive for AFB by direct microscopy, or a patient with one initial smear examination positive for AFB by direct microscopy and culture positive, or a patient with one initial smear examination positive for AFB by direct microscope and radiographic abnormalities consistent with active TB as determined by a clinician.
  Smear-negative pulmonary TB (PTB−): a patient having symptoms suggestive of TB with at least 3 initial smear examinations negative for AFB by direct microscopy, and (a) no response to a course of broad-spectrum antibiotics, and (b) again three negative smear examinations by direct microscopy, and (c) radiological abnormalities consistent with pulmonary tuberculosis, and (d) decision by a clinician to treat with a full course of anti-tuberculosis Or a patient whose diagnosis is based on culture positive for *M. tuberculosis* but three initial smear examinations were negative by direct microscopy.
  Treatment failure (*F*): a patient, while on treatment, was smear-positive at the end of the fifth month or later after commencing. Treatment failure also includes a patient who was initially sputum smear-negative but who becomes smear-positive during treatment.
  Treatment duration: the time recommended to treat/cure TB. It is 6 months for pulmonary TB and extrapulmonary TB without the involvement of the central nervous system (CNS) and 12 months for extrapulmonary TB which involves CNS TB Meningitis, TB Pericarditis, and 18 months and above for MDR-TB patients.
  Waiting time: the time spent by patients to receive drugs at the TB service unit.
  Distance: the distance (km) of the health facility from tb patient's home.
  Knowledge: patient's knowledge about the TB disease. It can be assessed by knowing the cause of TB, preventability, curability, and duration of TB treatment in months as well as knowing the consequences of stopping treatment.
  Patient's family support: a kind of support a patient received from family or friends during the treatment period. This support can be a reminder about the date of appointment, a reminder to take the drug or spiritual support.
  Perceived severity: patient's personal feeling of how serious the disease is. It can be assessed as very low if the patient perceives the disease as a not highly severe, medium if perceived as moderately severe, high if perceived as severe, and very high if perceived as highly severe.
  Perceived susceptibility: patient's feelings or perceptions about how vulnerable or risk he/she is to TB which is difficult to cure as a result of stopping taking TB medication. It can be measured as very low risk if a patient perceives him/herself as not highly at risk, medium if perceived as moderately at risk, high if perceived as at risk, and very high if perceived as highly susceptible or at risk.
  Perceived stigmatization: patient's personal feeling of being stigmatized/discriminated by other community members because of having TB disease.
  Perceived benefits: patient's belief about the benefits of taking the drug to cure his/her disease. It is measured as very low if a patient believes that taking doses will not totally cure him/her, medium if he/she believes that the drug will moderately/partially cure the disease, high if he/she believes the drug will cure him/her, and very high if he/she believes that the drug will cure him/her.
  Perceived barriers to treatment: patient's fear about what could stop him/her from taking TB drugs. It can be measured by assessing the barriers of adherence to TB treatment (drug side effects, drug burden, waiting time to get services, distance to a health facility, costs related to disease treatment, patient's privacy, and stigma). For health behavior (medication adherence) to be adopted, a person needs to believe that the benefits of taking medication outweigh the consequences of stopping the medication. This enables the barriers to be overcome and health behavior (overcoming nonadherence to TB medication) to be adopted.
  Smoking status: patient's smoking status during the entire treatment period of TB.
  Alcohol use: patient's alcohol drinking status during the entire treatment period of TB.
  Substance use: patient's use of the substance (chat, shisha, marijuana, and others) during the treatment period of TB.
  Treatment strategies during the initial phase: Patient's treatment strategy (home base and DOTS) during the initial phase of TB treatment.
  Health information: TB disease treatment information is provided to patients by the provider during the treatment period.
  Incentives/enablers: those things patients receive from health facilities and enable/motivate them to continue their treatment (e.g., transport, vouchers, reimbursement of transport, money, money, soap, food, and others).

## 3. Results and Discussion

### 3.1. Results

#### 3.1.1. Description of the Socio-Demographic and Economic Characteristics

**Figure 1:**
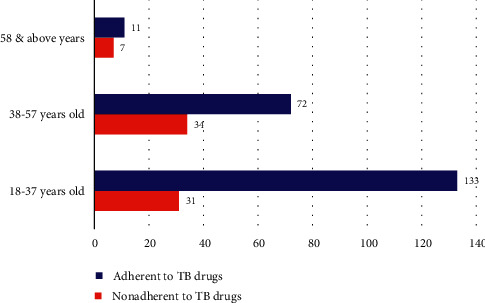
Patients age category versus TB-drug adherence category.

A semistructured interview was administered to 288 individuals, 72 cases, and 216 controls. In this study, the proportion of male participants in the cases (nonadhered TB patients) and controls (adhered TB patients) was 59.7% (43) and 63.4% (137) respectively (Table 2). The overall mean (standard deviation) age was 39.95 (13.4) years for cases and 35.4 (10.5) years for controls. Thirty-one (43.1%) cases and 133 (61.6%) controls were in the age range of 18 to 37 years (Figure 1). Fifty-one (70.8%) cases and 174 (80.6%) controls were married individuals. Among the study participants, twenty-nine (40.3%) cases and 81 (37.5%) controls were Nuer. Sixty-six (91.7%) cases and 195 (90.3%) controls were used to follow Christian religion. Forty-seven (65.3%) of the cases and 198 (91.7%) from the control group attended formal education. Seventeen (23.6%) cases and 120 (55.6%) controls were employees. The average monthly income of the study cases and controls were 1691.8 and 2128.5 in that order, while the overall mean income was 2019.34 Birr (Table 2).

#### 3.1.2. TB Patient Behavioral Factors

Forty-three (59.7%) of the cases and all controls knew the causative agent of TB is bacteria. Similarly, forty-eight (66.7%) of the cases and 215 (99.5%) controls also correctly responded that TB is a preventable disease. Sixty-seven (93.1%) cases and all controls believed that modern medications could cure TB. Forty-one (56.9%) cases and 206 (95.4%) controls believed in the advantage of regular medication to cure TB. The current study finding has revealed that forty-seven (65.3%) cases and 177 (81.9%) controls know the duration of TB treatment. The present study has also indicated that fifty-three (73.6%) cases and 212 (98.1%) controls know the consequence of interrupting TB medication. Forty-one (56.9%) cases and 4 (1.9%) controls missed their anti-TB drugs because of the feeling of no improvement sign after starting the medication. More than half (55.6%) of cases and 205 (94.9%) controls perceived TB as severe. Fifty-four (75%) cases and 46 (21.3%) controls did not feel that they would be susceptible to MDR-TB as a result of discontinuing treatment (Table 3).

**Figure 2:**
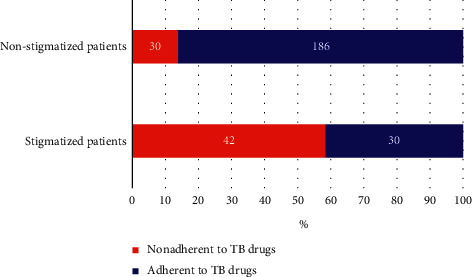
The proportion of TB patients who perceived stigmatized.

**Figure 3:**
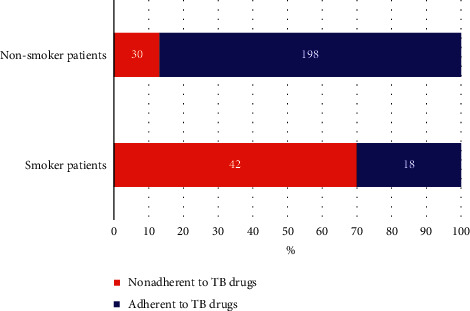
The proportion of TB patients who underwent smoking behavior.

Among the nonadherent patients to anti-TB drugs, 42 patients (58.3%) felt stigmatized (Figure 2) and used to smoke behavior (Figure 3).

Regarding the TB stigma, forty-two (58.6%) cases and 30 (13.9%) controls perceived that they were stigmatized because of developing TB. Forty-eight (66.7%) cases and 103 (47.7%) controls perceived that their privacy was not protected while receiving anti-TB treatment services. Forty-two (58.3%) cases and 18 (8.3%) controls reported smoking practices during the treatment schedule. Fifty-one (70.8%) cases and 11 (5.1%) controls used to drink regularly while on TB medication. Fifty-one (70.8%) and 203 (94%) controls reported that they had never used substances such as “chat,” marijuana and “hashish.” Concerning the patient's support (reminded medication time and appointment date) from family members or friends, fifty-one (70.8%) cases and 197 (91.2%) controls stated that they had received support. These and other behavioral factors of the study participants are displayed in Table 3.

#### 3.1.3. HCWs and Healthcare Provider-Related Factors

Forty-five (62.5%) cases and 3 (1.4%) controls missed their drugs during the intensive phase of TB treatment. Thirty-six (50%) cases and sixty-five (30.1%) controls were used to take home their anti-TB drugs during the initial phase of the anti-TB treatment. Thirty-eight (52.8%) cases and 77 (35.7%) controls had reported additional expenses related to TB tests. Forty-two (58.3%) cases and 3 (1.4%) controls had reported that lack of attention from HCWs (being so busy with other duties) was one of the reasons for missing medication. Forty (55.6%) cases and 6 (2.8%) controls pointed out that the drug burden (side effect) is one of the contributing factors for missing drugs. Seventy-one (98.6%) cases and 202 (93.5%) controls reported that they received insufficient information about TB treatment. Fifty-two (72.2%) cases and 55 (25.5%) controls revealed that they never received counseling service during the anti-TB treatment course.

Sixty-three (87.5%) cases and 183 (87.7%) controls showed that they failed to receive incentives (food, transport, voucher, money, or clothing) during the therapy. Fifty (69.9%) cases and 198 (91.7%) controls were stationed in an urban area for the entire period of treatment. Fifty-four (75%) cases and 207 (95.8%) controls lived nearby (less than 5 kilometres) to the healthcare provider/institution offering anti-TB medications and other services for treatment follow-up. Fifty-three (73.6%) cases and 120 (55.6%) controls were accustomed to using public transport (Minibus and Bajaj) services to access the anti-TB treatment services (Table 4).

#### 3.1.4. Bivariate Analysis of Socio-Demographic, Patient Behaviors, HCWs, and Healthcare Provider-Related Factors in Association with TB-Drug Nonadherence

The bivariate analysis results revealed that the age range from 38 to 57 years old, absence of formal education, unemployed, low income, rural residence, and lack of support from family or friends were associated with nonadherence to anti-TB treatment medications. On the other hand, the same analysis results indicated that having a lack of trust in the benefit of TB medications, lack of knowledge of the adverse effect of interrupting medications, perceiving TB as not severe, absence of perceived susceptibility to MDR-TB, inability to know the duration of anti-TB treatment, perceived stigma, perceived privacy breaches (lack of confidentiality and protection) and absence of counseling services during the anti-TB treatment period, smoking behavior, alcoholism/drunk, being drug users were associated with nonadherence. Furthermore, the bivariate analysis showed that taking anti-TB drugs to their home during the intensive phase, the additional expense incurred in relation to TB test and managing drug side effects, drug burden, living in a rural area, the distance of residence area from TB clinics (more than 5 kilometres), using public transport as a means of transport to access services hub, and absence of counseling during treatment was crudely associated with nonadherence to anti-TB treatment medications (Table 5).

#### 3.1.5. Factors Independently Associated with Nonadherence to Anti-TB Drug Treatment among TB Patients in Gambella Region

Five predictors were independently associated with nonadherence to anti-TB treatment medications among TB patients in four public health institutions of the Gambella regional state of Ethiopia. These independent variables were perceived stigma as a result of TB disease, lack of trust in the benefit of regular medications to get a cure for TB disease, perceived stigma, the perception of undermining TB as not a severe disease, absence of counseling services in the health institutions during the entire TB treatment course, and the patient's unhealthy behavior (smoking habit) during the treatment schedule. The adjusted odds ratios with the corresponding confidence intervals at 95% are given in Table 6.

### 3.2. Discussion

The Gambella Regional Health Bureau is striving to deliver to its full capacity on TB and HIV/AIDS diseases, with the existing manpower and resources at its hand. Above all, in Gambella, the key elements in TB control programs are to detect the disease and treat the cases to ensure the completion of their treatment for the inevitable cure [36]. However, regional TB control and treatment success is daunting with myriads of complex factors. Incomplete treatment leads to prolonged excretion of bacteria that may resist the drugs, which causes increased morbidity and mortality and makes fertile ground for the spread of the disease [43]. Hence, assessment of the status of treatment outcomes and finding out the specific determinant factors which contribute to this unsuccessful treatment course are imperative for the welfare of Gambella regional state and Ethiopia's TB management program. It is with this intention that the current study has been conducted among TB patients aged 18 years and above in Gambella regional state of Ethiopia. Although the current study findings have an indispensable attribute to the prospective TB control and treatment program in the Gambella region (Ethiopia); this study is not without its limitations, like many other studies. The inability to include pediatric TB patients, and failure to reach eight TB patients (two nonadherent cases and six adherent TB patients from the control group) in the most distant kebeles around the border with South Sudan were among the main limitations of this study.

Monitoring and evaluation play an important role in patient care and in assessing national programs and global response. Accurate recording and reporting of programmatic data inform the person in charge about the immediate output and outcomes of program services, as well as the larger impact of the resources invested into a program. Whenever services are adequately accessed, key activities are occurring promptly, and the expected results are achieved, then it is very likely that the overall goals will also be met. When monitoring and evaluation are running well, the healthcare providers and program managers provide tangible information on how many contacts were in a TB patient's household and how many of them were effectively evaluated. Similarly, how many people offered anti-TB treatment in an antiretroviral treatment clinic decided to take anti-TB medication, and how many of those starting anti-TB treatment continued it until the end [44].

The current study indicated that nonadherence to anti-TB treatment among TB patients, who were on DOTS in four public health facilities in Gambella region state, was influenced by patient behavioral factors and factors related to HCWs and healthcare providers (Table 6). Hence, this paper's findings solidly implicate the prominent determinants of nonadherence to anti-TB medications in the Gambella regional state of Ethiopia.

TB treatment discontinuation often leads to grave treatment outcomes, the development of MDR-TB, pro-extensively drug-resistant TB, and extensively drug-resistant TB; which are the current concerns of the East African, SSA regions in general, and Ethiopia in particular. In a bivariate analysis, numerous determinants have been found to have an association with nonadherence to anti-TB treatment. However, after various stages of model building and fit were established for its multivariable logistic regression, some exposure variables were found as the most significant predictors of nonadherence to anti-TB treatment in the Gambella regional state of Ethiopia. Thus, the study has pointed out that perceiving TB as nonsevere, and a lack of trust in the benefits of regular medications were independently associated with nonadherence to anti-TB drugs among TB patients. The possible justification for this could be the fact that perceiving the disease as not severe usually does not trigger or alert individuals to adopt health-seeking behavior (adhering to anti-TB medications). Unless patients perceive and believe it as a threat, it is difficult for them to understand the impact of the disease going to be imposed on their life. These fears or threats could be physical suffering, life-long impairment, financial burden, and/or loss of income as a result of contracting the disease.

Similarly, if patients do not believe in the benefits of taking action, the likelihood of adopting the new behavior (adhering to anti-TB medications) is unsatisfactory. Whenever a patient believes in the usefulness of this newly adopted behavior (adherence), it would likely decrease the risk of developing the notorious stages of the disease (disease progression and development of MDR-TB). Subsequently, it (newly adopted behavior) will not harm the individual TB patient and the safety of society as well. Perceived severity of TB and perceived benefit of regular medications were the constructs of the health belief model, which commonly influence healthy behavior (adherence to anti-TB treatment), as reported by previous research works from Indonesia [45], Isfahan [46], and Addis Ababa [47]. The current study's conclusive remarks in objecting that indicated previous scholarly work might emanate from the weakening TB control and prevention program follow-up in the Gambella region.

The study divulged that perceived stigma from others because of developing TB disease was a significant predictor of nonadherence to anti-TB drug treatment. As a result, those patients who were perceived as being stigmatized were 2.7 times more likely to be nonadherent compared to those who did not perceive stigma. Similar observations were documented by other scholars, who stated that TB was attached to stigmatization or rejection by the nearby community members [48, 49]. The possible explanation for the current study finding similarity with other previous reports might be related to the fear of stigma usually associated with HIV/AIDS comorbidity of the TB disease in the study area. Such denial behaviors of the social fabric and social isolation have a gateway for nonadherence to anti-TB drug treatment, eventually leading to poor treatment outcomes, MDR-TB development, death, and transmission of the more notorious TB disease-causing pathogens in the community.

Protecting the privacy of TB patients or keeping confidentiality is mandatory for TB patients. It is an essential issue in the TB control program, as the disease is associated with HIV/AIDS and unresolved social discrimination. In our extended detailed probing interview with TB patients from remote rural communities in the Gambella region of Ethiopia, the use of medically proven face masks (N-95) even made them feel socially discriminated (worthless). Alarmingly, this sense of perceived feeling continues under the COVID-19 disease pandemic, until the conclusion of this study. The HCW wearing a mask during the consultation hours with the patient creates the perception that the patient has a feeling of living with an incurable disease. With the increased thought of stigma associated with rough relationships and unnecessary words at the TB clinic from the HCWs, patients feel humiliated, disrespected, and perceived that the HCWs are conceited. This might frequently affect the trust between HCWs and patients, which has an impact on the willingness of the patient to return for follow-up consultations, visits, and anti-TB drug uptake. Thus, this act of denial could result in nonadherence, hence poor treatment realizations [50]. In the current study, patients who perceived that their privacy had not been protected were 7.98 times more likely to be nonadherent than those who perceived that their privacy was protected. Unfortunately, going to the wheel of patients in this regard might present a safety dilemma for the HCWs acting as a frontier on the DOTS window. However, regular delivery of health education through healthcare providers could do a lot of improvement in this context.

In addition, using the patient behavioral factor, the smoking habit has shown a significant association with nonadherence to anti-TB medication jobs. Being a smoker during anti-TB treatment was 15 times more likely to be nonadherent to medications than being a nonsmoker. This is consistent with the studies conducted elsewhere [51, 52]. According to the current paper results, patient-related factors contribute more significantly to nonadherence to anti-TB medications over nonpatient-related factors.

Likewise, healthcare provider-related factors also contributed to nonadherence to anti-TB drug treatment. Lack of counseling services during the treatment period had been significantly associated with nonadherence to anti-TB treatment. This clearly shows the importance of counseling as a key integral component of the anti-TB treatment regimen and protocols in African countries. Especially, anti-TB treatment medications go for a relatively long period that requires persistent follow-up, monitoring progress, and psychotherapy support for the patients to strictly adhere the patients to the treatment package. In support of the present study, many previous findings such as those from South Africa [53] and Bangladesh [54], indicated adequate institutional mandated counseling needs. These alleviate physical suffering, financial burden, nutritional needs, psycho-social (emotion entity) impairment as well as spiritual suffering of patients during the entire course of anti-TB drug treatment to bring efficient and effective adherence to medications.

Despite some research findings, which reported a positive association between the aforementioned predictors and nonadherence to anti-TB treatment drugs, our study did not show a significant association between lack of knowledge about TB and its duration of treatment, unlike the report from the Khartoum State of The Republic of Sudan [55]. The discrepancy could be because many development organizations of the United Nations, Western countries, and humanitarian organizations are extensively operating in the Gambella region, which might raise the knowledge of the community (TB causes, prevention, and cure) in the regional state. This could positively contribute to their awareness of the importance of anti-TB treatment, eventually enhancing their adherence to TB medications. Moreover, this study's results do not support a significant association between educational levels with nonadherence to anti-TB treatment. In this regard, a couple of research study papers are not in line with our study; such as those conducted in the Brazilian Amazon area [56] and Equatorial Guinea [57] were among others. The disagreement between the previously reported findings and the present study could be because, in the current study, most of the study subjects had formal education which might also contribute to their knowledge of the disease treatment and could be a protective reason for nonadherence to anti-TB medications.

In this particular study, like many other study reports, income has not been found to associate with nonadherence to anti-TB drug medications. The previous study in Kenya [58] did not report this relationship, where TB disease was positively associated with poverty and low-income status of the TB patient's household. Our study findings disagree with the notion that nonadherence to anti-TB drug medications is mostly practiced by the poor section of the community, hard-to-reach people, and those who did not afford the transportation costs for accessing anti-TB treatment centers from distant areas. Besides, the distance of patient residence area to anti-TB control and treatment service unit was not independently predicted nonadherence to anti-TB drug medications. The probable reason for this could be the proximity (less than 5 kilometres) of the TB clinic to the majority of the TB patients included in the current study, at least through rental mechanisms for the duration of treatment. This result goes in line with the previous report from Khartoum in 2016 [55]. Another group of researchers from Nepal [59] and Ethiopia [60] have also documented similar findings that proximity to TB clinics was not a risk factor for nonadherence to TB medications.

Regarding the barriers to access to anti-TB treatment service units; particularly the transport means (using public transport) were not significantly correlated with the interruption of anti-TB drugs in the present study. The reasons may not differ from that of the income level of the TB patients explained above and might indicate their ability to afford costs related to the transport to reach the TB clinic. Furthermore, the current study did not show a significant association between the absence of family support and nonadherence to anti-TB medications. This later narrative is not in agreement with the previous study [55] reported from Sudan, where the current study TB patients received support from their families which helped them to comply with uninterrupted anti-TB medications.

The bottom line is that the study has addressed various determinants of nonadherence to anti-TB treatment including patient behavioral components using the constructs of the health belief model, a health model commonly designed to execute the existing gap and subsequently used as an index for improving anti-TB treatment-seeking behavior of patients. Unlike the current study, most previous studies on nonadherence to anti-TB medications have exclusively dealt with socio-economic-related factors. Another strength and positive attribute of the present study is that the recall bias was minimized by reviewing the patient medical records and by cross-checking some variables that might intrude recall bias or impart recall difficulty. As a result, the duration of anti-TB treatment, anti-TB treatment phases, and months where a patient missed the drugs were efficiently retrieved.

Finally, the solutions to the problems in TB management are protecting patients during the treatment cycle and completion of the treatment period. However, the negative outcomes of not adhering to the treatment are extensive. Thus, recognizing the determinant factors related to the nonadherence to anti-TB drugs, medication behavior of the patients is an important key issue in the reduction of some outcomes; such as the development and weird transmission of drug-resistant and extensively drug-resistant versions of microbes, retreatment costs, disability and death.

## 4. Conclusions

Nonadherence to antituberculosis medications conspicuous or suboptimal, remains a substantial TB control problem in Gambella regional state of Ethiopia, as it is dwindling nationally reported success avenue awry. Such anti-TB drug discontinuations sustain infectiousness in the whole community and facilitate the selection of multidrug-resistant (MDR) pathogens. The independent predictors and determinant factors of nonadherence to anti-TB treatment in Gambella regional state are perceived stigma as a result of TB disease, lack of trust in the benefit of regular medications, perceived stigma, the perception of undermining TB (as a mild illness), absence/irregularity of counseling services by healthcare providers in the region, and the patient's unhealthy behavior (smoking habit) during the treatment course. Hence, the national TB policy formulators on TB control programs together with the international development organizations (funded by the treasury of Westerners) need to take early corrective tuning measures. Moreover, the regional health office should strengthen health education among the community and offer continuous training to its workers. The healthcare workers' training should at least entail actionable biomedical ethics and privacy principles. Still equally much has to be done in the region on the stigma associated with HIV/AIDS, after remarkable remission in the rest of the world over the last 5 decades, as it is still one of the sole contributors to the humiliation linked to anti-TB medication adherence in Gambella.

## Figures and Tables

**Table 1:** Proportionate sample size allocation to four health facilities in the Gambella region.

Health facilities	Patients load in 2018 3^rd^ quarter (*P*)	Controls per case	*n*	(*c*^*∗*^*n*)
Gambella hospital	497	3	44	132
Punyido health center	124	3	11	33
Kuergeng health center	136	3	12	36
Nyinengang health center	56	3	5	15
Subtotal	813	3	72	216
Total sample size (*n* + *cn*)			**288**	

**Table 2:** Sociodemographic and economic factors among TB patients about TB-drug nonadherence in Gambella region.

Socio-demographic characteristics		Nonadhered number (%)	Adhered number (%)
Sex	Male	43 (59.7)	137 (63.4)
	Female	29 (40.3)	79 (36.6)
Marital status	Married	51 (70.8)	174 (80.6)
	Unmarried	21 (29.2)	42 (19.4)
Education	Formal education	47 (65.3)	198 (91.7)
	No formal education	25 (34.7)	18 (8.3)
Occupation	Employed	17 (23.6)	120 (55.6)
	Unemployed	55 (76.4)	96 (44.4)
Religion	Christian	66 (91.7)	195 (90.3)
	Muslim	6 (8.3)	21 (9.7)
Ethnicity	Nuer	29 (40.3)	81 (37.5)
	Agnuwuak	20 (27.8)	51 (23.6)
	Others	23 (31.9)	84 (38.9)
Income (birr)	Earn <501 birr^*ψ*^	34 (47.2)	35 (16.2)
	Earn 501 or more birr^*ψ*^	38 (52.8)	181 (83.8)

^*ψ*^ = the mean income earned monthly in Ethiopia currency (Birr).

**Table 3:** TB patient behavioral factors about TB-drug nonadherence in Gambella region.

Patient's behavioral factors		Nonadhered number (%)	Adhered number (%)
The cause of TB	Bacteria	43 (59.7)	216 (100)
	Do not know	29 (40.3)	0 (0.0)
TB preventable	Yes	48 (66.7)	215 (99.5)
	No	24 (33.3)	1 (0.5)
Modern medications cure TB	Yes	67 (93.1)	216 (100)
	No	5 (6.9)	0 (0.0)
The benefit of regular medication	Cure	41 (56.9)	206 (95.4)
	Not cure	31 (43.1)	10 (4.6)
Duration of TB treatment	Knew	47 (65.3)	177 (81.9)
	Did not know	25 (34.7)	39 (18.1)
A consequence of missing drugs	Yes	53 (73.6)	212 (98.1)
	No	19 (26.4)	4 (1.9)
Reasons for drug interruption	No improvement	41(56.9)	4 (1.9)
	Embarrassment	31 (43.1)	2 (0.9)
	No personal reasons	0 (0.0)	210 (97.2)
Severity of TB	Severe	40 (55.6)	205 (94.9)
	Not severe	32 (44.4)	11 (5.1)
Susceptibility to MDR-TB	Susceptible	18 (25)	170 (78.7)
	Not susceptible	54 (75)	46 (21.3)
Privacy	Protected	24 (33.3)	113 (52.3)
	Unprotected	48 (66.7)	103 (47.7)
Alcohol habit	Nondrunker	21 (29.2)	205 (94.9)
	Drunker	51 (70.8)	11 (5.1)
Substance abuse	Nonuser	51 (70.8)	203 (94)
	User	21 (29.2)	13 (6)
Family support	Received	51 (70.8)	197 (91.2)
	Not received	21 (29.2)	19 (8.8)

**Table 4:** HCWs and healthcare provider-related factors in relation to TB-drug nonadherence in Gambella region.

Exposure factors		Nonadhered number (%)	Adhered number (%)
Missing dose in the treatment phase	Never missed anti-TB drugs	0 (0.0)	210 (97.2)
	Missed drugs in the initial phase	45 (62.5)	3 (1.4)
	Missed drugs at continuation phase	27 (37.5)	3 (1.4)
Treatment strategy	DOTS	36 (50)	151 (69.9)
	Home-based	36 (50)	65 (30.1)
Reasons for TB-related cost	No incurred cost	31 (43)	111 (51.4)
	For TB test	38 (52.8)	77 (35.7)
	Managing drug side effects	3 (4.2)	28 (13)
Health care factors for missing drugs	No reasons	12 (16.7)	210 (97.2)
	Provider being busy	42 (58.3)	3 (1.4)
	Waiting time	18 (25)	3 (1.4)
Disease/treatment factors to missed drug	No reasons	32 (44.4)	210 (97.2)
	Drug burden and side effects	40 (55.6)	6 (2.8)
Health information	Received	71 (98.6)	202 (93.5)
	Not received	1 (1.4)	14 (6.5)
Incentives	Received	9 (12.5)	33 (15.3)
	Not received	63 (87.5)	183 (87.7)
Residence	Urban	50 (69.4)	198 (91.7)
	Rural	22 (30.6)	18 (8.3)
Distance to clinic	<5 kms	54 (75)	207 (95.8)
	≥5 kms	18 (25)	9 (4.2)
Transport means	On foot	19 (26.4)	96 (44.4)
	Public transport	53 (73.6)	120 (55.6)
Got counseling	Yes	20 (27.8)	161(74.5)
	No	52 (72.2)	55 (25.5)

**Table 5:** Bivariate analysis of socio-demographics, patient's behavior, and HCWs and healthcare provider-related factors are associated with nonadherence to anti-TB medications in Gambella.

Socio-demographic characteristics		Nonadhered number (%)	Adhered number (%)	OR	95% CI	*P* value
Age (years)	18–37	31 (43.1)	133 (61.6)	1		
	38–57	34 (47.2)	72 (33.3)	2	1.15–3.56	0.014^*∗*^
	≥58	7 (9.7)	11 (5.1)	2.73	0.98–7.61	0.055
Educational status	No formal education	25 (34.7)	18 (8.3)	5.85	2.95–11.6	0.001^*∗*^
	Formal education	47 (65.3)	198 (91.7)	1		
Occupation	Employed	17 (23.6)	120 (55.6)	1		
	Unemployed	55 (76.4)	96 (44.4)	4	2.21–7.42	0.001^*∗*^
Income	<501.00 Birr/Mon	34 (47.2)	35 (16.2)	4.63	2.57–8.33	0.001^*∗*^
	≥501.00 Birr/Mon	38 (52.8)	181 (83.8)	1		
Residence	Urban	50 (69.4)	198 (91.7)	1		
	Rural	22 (30.6)	18 (8.3)	4.84	2.4–9.71	0.001^*∗*^
Family support	Received	51 (70.8)	197 (91.2)	1		
	Not received	21 (29.2)	19 (8.8)	4.269	2.14–8.54	0.001^*∗*^

*Patient behavioral factors*						
Regular medication benefit	Cure	41 (56.9)	206 (95.4)	1		
	No cure	31 (43.1)	10 (4.6)	15.58	7.1–34.2	0.001^*∗*^
Duration of TB treatment	Knew	47 (65.3)	177 (81.9)	1		
	Not knew	25 (34.7)	39 (18.1)	.053	.017–0.161	0.001^*∗*^
Impact of missing	Yes	53 (73.6)	212 (98.1)			
	No	19 (26.4)	4 (1.9)	2.4	1.3–4.38	0.004^*∗*^
Severity of TB	Severe	40 (55.6)	205 (94.9)	1		
	Not severe	32 (44.4)	11 (5.1)	14.9	6.9–32	0.001^*∗*^
Susceptibility to MDR-TB	Susceptible	18 (25)	170 (78.7)	1		
	Not susceptible	54 (75)	46 (21.3)	11.1	5.9–20.7	0.001^*∗*^
Stigmatization	No stigma	30 (41.7)	186 (86.1)	1		
	Stigmatized	42 (58.3)	30 (13.9)	7.6	4.1–14.2	0.001^*∗*^
Privacy	Protected	24 (33.3)	113 (52.3)	1		
	Unprotected	48 (66.7)	103 (47.7)	2.2	1.3–3.8	0.001^*∗*^
Smoking habit	No smoking	30 (41.7)	198 (91.7)			
	Smoker	42 (58.3)	18 (8.3)	15.4	7.7–30	0.001^*∗*^
Alcohol habit	Nondrunk	21 (29.2)	205 (94.9)	1		
	Drunker	51 (70.8)	11 (5.1)	45.3	20.5–99.9	0.001^*∗*^
Substance abuse	Nonuser	51 (70.8)	203 (94)	1		
	User	21 (29.2)	13 (6)	6.4	3–13.7	0.001^*∗*^

*HCWs and healthcare provider-related exposure factors*						
Treatment strategy	DOTS	36 (50)	151(69.9)	1		
	Home based	36 (50)	65 (30.1)	2.323	1.33–4	0.002^*∗*^
Reasons for related cost	Not incurred	31 (43)	111 (51.4)	1		
	For TB test	38 (52.8)	77 (35.7)	1.767	1.0–3.0	0.045^*∗*^
	Manage drug side effect	3 (4.2)	28 (13)	0.384	0.1–1.3	0.135^*∗*^
Disease treatment factors	No reasons	32 (44.4)	210 (97.2)	1		
	Drug burden	40 (55.6)	6 (2.8)	43.75	17–111	0.001^*∗*^
Residence	Urban	50 (69.4)	198 (91.7)	1		
	Rural	22 (30.6)	18 (8.3)	4.8	2.4–9.7	0.001^*∗*^
Distance	<5 kms	54 (75)	207 (95.8)	1		
	≥5 kms	18 (25)	9 (4.2)	7.67	3.2–18	0.001^*∗*^
Transport	On foot	19 (26.4)	96 (44.4)	1		
	Public transport	53 (73.6)	120 (55.6)	2.2	1.2–4.0	0.008^*∗*^
Counseled	Yes	20 (27.8)	161 (74.5)	1		
	No	52 (72.2)	55 (25.5)	7.6	4.18–13.86	0.001^*∗*^

*Note.* (^*∗*^) indicates (*P* value less 0.25), 1 indicates the reference variable; OR = crude odds ratio, CI = confidence interval at 95% significance level; mon = month.

**Table 6:** Factors independently associated with nonadherence to anti-TB treatment among TB patients in Gambella region.

Exposure variables		AOR	CI at 95%	*P* value
Perceived privacy	Protected (reference)	1		
	Unprotected	1.2	0.53–2.86	0.63
Perceived stigma	Not stigmatized	1		
	Stigmatized	2.7	1.1–6.6	0.03
The benefit of regular medication	Cure	1		
	No cure	6.8	1.8–24.9	0.04
Perceived severity of TB	Severe	1		
	Not severe	8.38	2–34.6	0.03
Perceived susceptibility to MDR-TB	Yes	1		
	No	1.2	0.5–2.99	0.65
Got counseling	Yes	1		
	No	35.5	10.3–122	0.001
Smoking habit	No-smoking	1		
	Smoker	10.9	4–29.4	0.001

*Note.* 1 indicates the reference variable, AOR = adjusted odds ratio, CI = confidence interval at 95% significance level.

## Data Availability

The datasets used and/or analyzed during the current study are available from the corresponding author.

## Abbreviations

AIDS: Acquired immunodeficiency syndrome
CNS: Central nervous system
CSA: Central Statistical Agency of Ethiopia
DOTS: Directly observed treatment short course
EPTB: Extrapulmonary tuberculosis
FDRE: Federal Democratic Republic of Ethiopia
HIV: Human immunodeficiency virus
IRB: Institutional review board
MDGs: Millennium development goals
MDR-TB: Multidrug-resistant tuberculosis
PTB: Pulmonary tuberculosis
RR-TB: Rifampicin resistant tuberculosis
SDGs: Sustainable development goals
SNNPR: Southern Nations, Nationalities and Peoples' Region
SPSS: Statistical Package for Social Science
TB: Tuberculosis
WHO: World Health Organization
XDR-TB: Extensively drug-resistant tuberculosis.
